# Exploring CCL11 in breast cancer: unraveling its anticancer potential and immune modulatory effects involving the Akt-S6 signaling

**DOI:** 10.1007/s00432-023-05600-6

**Published:** 2024-02-02

**Authors:** Xiao Chen, Chenxu Meng, Xinyu Wang, Zanhui Wu, Xinyue Sun, Chenyu Sun, Lu Zheng, Wanwan Li, WenJun Jia, Tong Tang

**Affiliations:** 1 Department of Thyroid and Breast Surgery, The Second Affiliated Hospital of Anhui Medical University, Hefei, China; 2 Anhui Medical University, Hefei, China

**Keywords:** CCL11, Breast cancer, Tumor microenvironment, Immune infiltration, Biomarker

## Abstract

**Background:**

CCL11, a chemokine known for recruiting immune cells to the tumor microenvironment (TME), has an unclear role in the context of its expression, patient prognosis, and the presence of tumor-infiltrating immune cells (TILs) in breast cancer.

**Methods:**

The expression of CCL11 in invasive breast cancer (BRCA) was analyzed using TCGA database. Survival curve and Cox regression analysis determined the potential of CCL11 as an independent prognostic indicator. GSEA performed functional analysis on genes related to CCL11. CIBERSORT algorithm quantified the infiltration level of immune cells with varying CCL11 expression. Lastly, the correlation between CCL11 expression and anticancer drug sensitivity was examined. Immunohistochemistry (IHC) and qRT-PCR confirmed CCL11 expression in clinical tissue samples. The anti-tumor efficacy of CCL11 was investigated using CCK-8, plate formation, transwell assay, and Western blot.

**Results:**

CCL11 expression was elevated in BRCA tumor tissues compared to adjacent normal tissues. Recurrence-free survival (RFS) was longer in patients with high expression of CCL11. Enrichment and co-expression analyses revealed CCL11's association with numerous immune-related signaling pathways and genes. Validation studies confirmed high CCL11 expression in breast cancer tissues. In vitro experiments substantiated CCL11's anticancer effects in BRCA.

**Conclusion:**

CCL11 expression correlates with immune cell infiltration in breast cancer, indicating its potential as a prognostic biomarker for BRCA.

**Supplementary Information:**

The online version contains supplementary material available at 10.1007/s00432-023-05600-6.

## Introduction

Breast cancer represents the most prevalent malignancy among women globally. The 2020 report from the International Cancer Research Center indicates that breast cancer surpasses lung cancer in mortality, rendering it the leading cause of cancer-related deaths worldwide (Sung et al. 2020). Breast cancer treatment encompasses surgery, chemotherapy, radiotherapy, targeted therapy, endocrine therapy, and increasingly, immunotherapy (Yu et al. 2017).

Immunotherapy works by initiating or restoring the body's own anti-tumor immunity via activation or inhibition of pathways directly or indirectly tied to the immune response (Kennedy and Salama 2020). The tumor microenvironment (TME) is the primary site of this endogenous anti-tumor immunity, where immune cells execute functions that enable effective immunotherapy (Azizi et al. 2018). CD4 + T cells exert direct cytolytic effects on tumors and bolster innate immune cell activity via IFN-γ production. CD4 + T cells prime dendritic cells (DCs) for effective antigen presentation to CD8 + T cells and secrete IL-2 to maintain CD8 + T cell memory, fostering enduring cytotoxic responses. CD8 + T cells secrete granzyme B, IFNγ, and TNFα, essential for tumor cell eradication (Caserta et al. 2012). DCs are essential in initiating anti-tumor immune responses by bridging innate and acquired immunity and presenting tumor-derived antigens to T cells (Cunha et al. 2014). Macrophages are recruited into malignant tumors through chemokines secreted by cancer and stromal cells. They secrete various tumor-promoting factors, such as vascular endothelial growth factor (VEGF), IL-10, and TGF-β, which foster angiogenesis, immune evasion, and consequently, tumor invasion and metastasis (Solinas et al. 2009). Neutrophils, key immune cells within tumors, exhibit phenotypic plasticity with potential anti-tumorigenic (N1) or protumorigenic (N2) roles (Mishalian et al. 2017). B cells perform anti-tumor activities such as directly killing tumor cells, inducing Th1 responses, and generating Treg cells (Mahmoud et al. 2012).

The TME primarily comprises tumor-infiltrating immune cells (TILs) recruited by chemokines, which can either promote or inhibit tumor growth (Wang et al. 2021). Chemokines facilitate the infiltration of immune cells into the TME, recruiting specific immune-related cell types and exerting direct and indirect effects on tumor cells (Mahmoud et al. 2012). Importantly, chemokines not only recruit specific cells but also mediate signal transduction (through ligand–receptor interactions), enhancing cancer cell proliferation, migration, invasion, and drug resistance. Cancer cells utilize chemokines to recruit specific cells and reprogram the microenvironment, ensuring conditions favorable for their survival (Nagarsheth et al. 2017). Thus, chemokines are integral to the TME composition (Vicari and Caux 2002).

CCL11, also known as eotaxin, a member of the CC chemokine subfamily, recruits eosinophils to the TME, playing an anti-tumor role (Thomas et al. 2019). CCL11 is implicated in inflammation, significantly contributing to skin and lung inflammatory processes and fibrosis, with eosinophils playing a major role (Sugaya 2015). Studies on CCL11's role and relevance in tumors are limited compared to other chemokines. Notably, CCL11 has been found to be upregulated in colorectal cancer (CRC), breast cancer, and oral squamous cell carcinomas (OSCC) (Thomas et al. 2019; Reichman et al. 2019; Lorena et al. 2003). CCL11 also regulates the proliferation and invasion of ovarian carcinoma cells via CCR3 (Levina et al. 2009). Moreover, CCL11 may act in an autocrine manner in anaplastic large cell lymphoma (ALCL) to promote tumor growth (Sugaya 2015). However, it remains unclear whether CCL11 influences tumor progression via TILs in breast cancer's TME.

TME can either promote or prevent the development, expansion, and metastasis of tumor cells. In this study, we investigate the immunological roles and immune cell profiles linked to CCL11 expression in patients with BRCA. Furthermore, we evaluated the expression profile and clinical significance of CCL11 in patients with BRCA using multiomics data and clinical samples. Various immunological algorithms have been used to analyze the immune environment involved in CCL11 expression. The tumor-inhibiting effects of CCL11 were validated in vitro assays. The objective of this study was to investigate the biological significance of CCL11 in TME of breast cancer.

## Materials and methods

### Ethical approval

This study was approved by the Institutional Research Ethics Committee of The Second Affiliated Hospital of Anhui Medical University and written informed consent was obtained from all patients under the Ethics approval No. AHMU2ND-2021-23. The present study evaluated 120 patients who underwent radical mastectomy at The Second Affiliated Hospital of Anhui Medical University (Hefei, China) between January 2017 and December 2019. We included patients with pathological types of invasive breast carcinoma, including invasive tubular carcinoma and invasive lobular carcinoma. Patients who received chemotherapy or radiotherapy before surgery were excluded from analyses. Our study complied with the Declaration of Helsinki.

### Data collection and differential expression analysis

RNA sequencing, somatic mutations, and related clinical data were downloaded from The Cancer Genome Atlas (TCGA) (https://www.cancer.gov/about-nci/organization/ccg/research/structural-genomics/tcga). CCL11 expression was evaluated in tumors and normal tissues using the downloaded data and CCL11 expression levels that were compared between cancer samples and matched pan-cancers standard samples. Transcribed values were log2 transformed using the "limma" package in the R language. All data involved in this study were acquired and applied in accordance with the database publication guidelines and data access policies.

### Prognosis analysis

Survival differences between high and low CCL11 groups were evaluated using Kaplan–Meier Plotter (http://kmplot.com/analysis/index.php?p=background). Overall survival (OS), relapse-free survival (RFS), distant metastasis-free survival (DMFS), and post-progression survival (PPS) were used to assess the prognostic value of CCL11 in breast cancer. We conducted univariate and multivariate Cox regression analyses to ascertain the clinical significance of CCL11 expression.

### Construction of protein interaction (PPI) network and analysis of functional enrichment

The PPI network of top50 protein interacting with CCL11 is from SRTING database (https:/cn.string db.org). The results were visualized by Cytoscape. Gene Ontology (GO) and Kyoto Encyclopedia of Genes and Genomes (KEGG) (Ogata et al. 1999; Kanehisa 2019; Kanehisa et al. 2022) were used to evaluate the potential function of CCL11 through R package "clusterProfiler"(Yu et al. 2012).

### Analysis of co‑expression genes

The link lookup module of LinkedOmics database was used to perform differential expression gene analysis of CCL11 in BRCA. Pearson correlation coefficients were used for statistical analysis. The results were presented in volcano map and heat map.

TISIDB (cis.hku.hk/TISIDB/index.php), a database for studying the interaction between tumor and immunity, was used to perform gene co-expression analysis on CCL11, including immune activator, immunosuppressant, chemokine, chemokine receptor, and MHC gene.

### Analysis of immune infiltration

CIBERSORT (Chen et al. 2018) was used to calculate the degree of immune cell infiltration in the BRCA queue in TCGA. Wilcoxon signed rank test was applied to analyze the differences in tumor-infiltrating immune cells between different groups. The ESTIMATE algorithm was used to calculate the immune and matrix scores between two groups (Li et al. 2020). We use TIMER 2.0 database (timer.cistrome.org) to study the association of CCL11 expression with immune cell infiltration.

### Analysis of drug sensitivity

Immunophenotypic scores (IPS) were used to predict the response to immunotherapy in CCL11 low- and high-expression groups. A higher IPS score indicates greater immunogenicity. IPS for TCGA-BRCA patients were obtained from the Cancer Immunome Atlas (TCIA) (https://TCIA.at/home). RNA sequencing expression profiles and corresponding clinical information for CCL11 in BRCA were downloaded from TCGA dataset. Drug sensitivity analysis was conducted using the “pRRophetic” package and “ggplot2″ tools (Geeleher et al. 2014).

### Immunohistochemistry (IHC) staining

The tumor tissue was fixed and embedded in paraffin. After that, the samples were cut into 3-µm-thick slices and stained with immunohistochemistry. The primary antibodies used in the study were as follows: anti-CCL11 (1:500; Abcam; ab203586), CD4 (1:200; Abcam; ab183685), and Foxp3 (1:2000; Abcam; ab75763). The primary antibody was incubated at 4℃ overnight. After washing with PBS, secondary antibody was added and incubated at room temperature for 1 h. With (DAB) color development, hematoxylin inhibits the nucleus. Finally, the staining quantity and intensity were comprehensively analyzed. The IHC results were evaluated using the intensity score. IHC scores were determined by multiplying the score for staining intensity (0, negative; 1, weak; 2, moderate; 3, strong) with the score or positive area (0, less than 5%; 1.5–25%; 2.26–50%; 3.51–75%; 4, greater than 75%). Scores of 0 to 7 were considered as a low expression and scores of 8–12 as a high expression. The scoring criteria referred to the following literature (Shan et al. 2013; Tuo et al. 2022).

### Quantitative real-time polymerase chain reaction (qRT-PCR)

Total RNA was extracted by TRIzol (Invitrogen, USA). The extracted RNA is reverse transcribed into cDNA. QRT-PCR kit (QR0100-1KT, Sigma-Aldrich, USA) was used to quantify the extracted RNA. GAPDH was taken as the internal parameter. The relative expression was quantified by 2 − ∆∆Ct method. Primer information is shown in Supplementary Table 1.

### Cell culture

Human breast cancer cell lines (MCF-7 and MDA-MB-231) were bought from the American Type Culture Collection (ATCC). All cells were cultured in DMEM medium with 10% fetal bovine serum and 100 × penicillin–streptomycin solution. All cells were cultured at 5% CO_2_ and 37 °C. Recombinant Human CCL11 (JN0318, Baiaoleibo, Beijing) was added to the cell line and continued to culture.

### Cell proliferation assay

Cells were inoculated into 96-well plates. Cell viability was calculated by CCK-8 system after 24, 48, and 72 h. The optical density (OD) value of each hole was measured at 450 nm (BioTek USA).

### Colony formation assay

Cells were cultured in 6-well plates for 2 weeks. 500 cells per well were cultured until colony formation. The formed colonies were fixed with 4% paraformaldehyde (PFA) for 30 min and stained with 0.5% crystal violet for 5 min. The number of clones formed (more than 50 cells per clone) was manually measured and photographed.

### Transwell

For invasion assay, the top chamber was pre-coated with Matrigel. Next, the cells were added culturing in a serum-free medium. In the meantime, RPMI-1640 medium with 10% FBS was put in the lower chamber. After that, cells that remained in the upper chamber were removed. Invaded cells were finally stained with crystal violet and observed. For migration assay, the upper chamber was not coated with Matrigel, and the remaining procedures were similar to those in the invasion assay.

### Western blot

Cellular proteins were extracted by mixing phenyl methane sulfonyl fluoride (PMSF) with radioimmunoprecipitation test buffer (Beyotime, Jiangsu, China). The detached proteins were then loaded into 10% SDS-PAGE gel for analysis. The isolated proteins are transferred to PVDF membranes (Bio-Rad, CA, USA). After blocking with 5% buttermilk, the cells were incubated overnight at 4 °C with anti-Akt (1:1000), p-AKT (1:1000), S6(1:1000), p-S6(1:1000), and internally controlled anti-GADPH (1:1000) (Abcam, UK) primary antibodies. The membrane was washed with TBST and incubated for 1 h with the addition of secondary antibody. Finally, Western blot exposure was performed using Pierce ECL (Thermo Fisher Scientific, USA). Image J is used for data analysis.

### Statistical analysis

R (version 4.2.1) was used for bioinformatics analysis. The algorithm used and the R package are written in the methods section. Statistical analyses were conducted by GraphPad Prism 7.0 (GraphPad Software Inc., CA, USA) and SPSS 21.0 (IBM, SPSS, Chicago, IL, USA). Data are expressed as the mean ± S.D (Standard Deviation). All continuous data were tested for normality and compared between multiple groups using one-way analysis of variance. Two-way ANOVA was used for multiple group comparisons, and unpaired two-tailed t-tests for two-group comparisons. Univariate and multivariate Cox proportional hazards regression models were used to identify factors associated with survival. Significant statistical differences between groups were indicated as follows: *p < 0.05; **p < 0.01; ***p < 0.001.

## Results

### Correlation between CCL11 expression and prognosis in BRCA

**Fig. 1:**
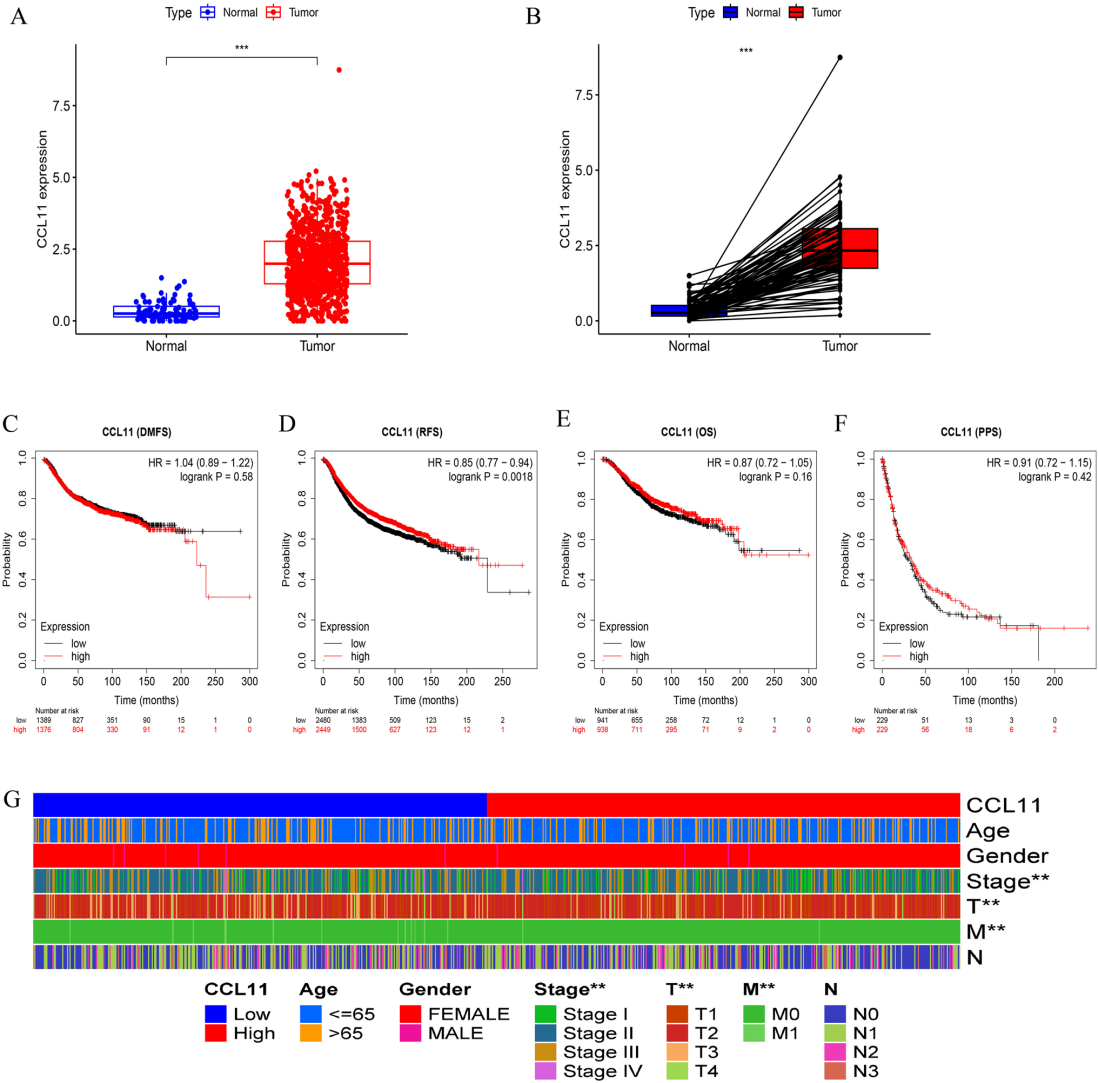
**A**, **B** Expression of CCL11 in tumor tissues and normal tissues of breast cancer from TCGA database; **C**–**F** OS, RFS, PFS, and DSS in breast cancer patients; **G** correlation analysis between CCL11 and different pathological features in breast cancer. *OS* overall survival, *RFS* relapse-free survival, *PPS* post-progression survival, *DMFS* distant metastasis-free survival

We analyzed the dataset of BRCA in TCGA database which included 1097 BRCA tissues and 114 adjacent tissues. Our analysis revealed that CCL11 expression is markedly elevated in BRCA tissues compared to adjacent non-cancerous tissues (Fig. 1A, B). All breast cancer samples were categorized into high-expression and low-expression groups using the median expression level as a threshold. Analyzing survival data using Kaplan–Meier survival analysis indicated that higher levels of CCL11 were significantly associated with better RFS (Fig. 1C–F). Subsequently, CCL11 was significantly correlated with the pathological features of BRCA, including Stage, T, and M (Fig. 1G).

**Fig. 2:**
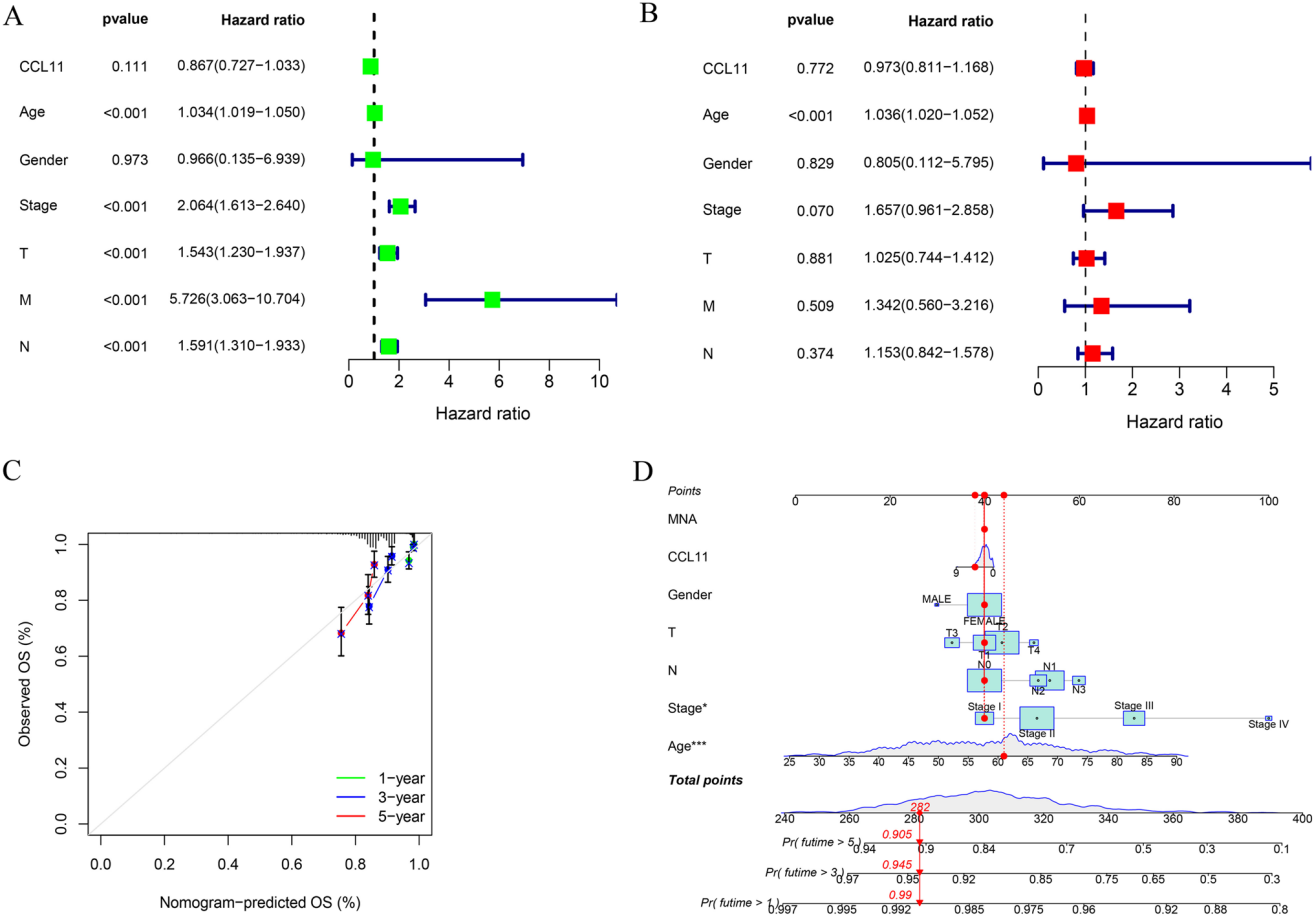
**A** Univariate Cox regression analysis of CCL11 in breast cancer; **B** multivariate Cox regression analysis of CCL11 in breast cancer; **C** the prognostic nomogram with CCL11 and clinical variables; **D** the 1-, 3-, and 5-year calibration plots demonstrated the performance of the nomogram

Univariate and multivariate Cox regression analyses were performed to determine independent prognostic factors. Univariate Cox regression analysis showed that age, stage, T, M, N, and CCL11 were closely related to survival (Fig. 2A). Multivariate analysis indicated that age was a significant predictor of survival (Fig. 2B). The prognosis histogram with CCL11 and clinicopathological features is shown in Fig. 2C. The 1-year, 3-year, and 5-year calibration charts illustrate the accuracy of the bar chart (Fig. 2D).

### The establishment of PPI network and enrichment analysis of CCL11 in BRCA

**Fig. 3:**
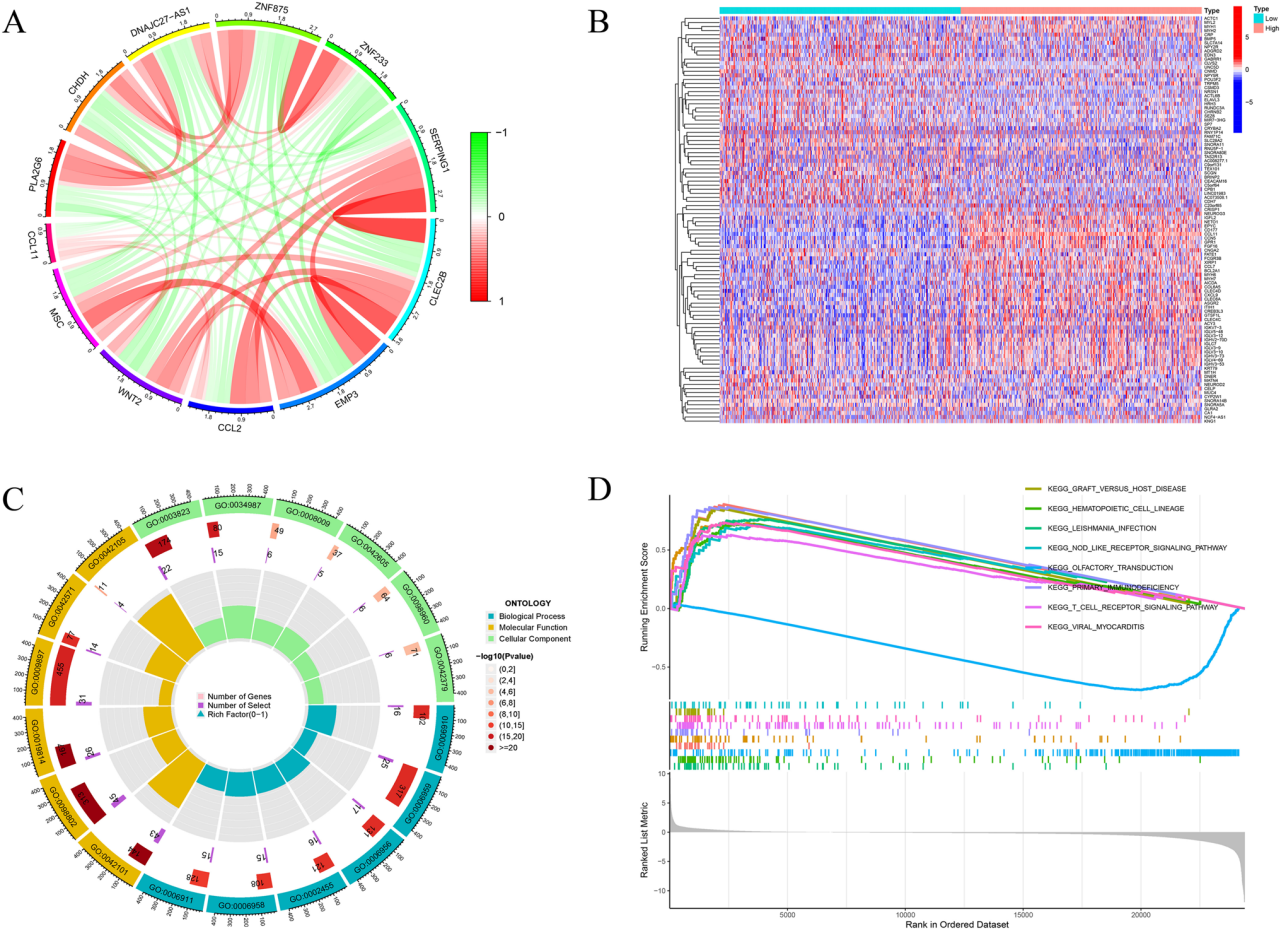
**A** Co-expressed genes significantly associated with CCL11 were shown as correlation circle plots; **B** the top 50 upregulated DEGs and top 50 downregulated DEGs are displayed in the heat map (red, upregulated genes; blue, downregulated genes); **C** the circle graph of enrichment analysis; **D** the signaling pathways that were CCL11-related differentially expression genes activated in breast cancer through GSEA

The complex network map established in the STRING database represents the interaction of CCL11 protein and its functionally related proteins (Supplementary Fig. 1). The circle chart shows 10 co-expressed genes that are significantly positively or negatively associated with CCL11 (Fig. 3A). Breast cancer samples were divided into high-expression group and low-expression group according to the median expression level of CCL11. DEGs analysis was compared between the two groups with |log2(Fold Change) |> 1 and adjusted *p* < 0.05. The top 50 upregulated genes and top 50 downregulated genes are shown in Fig. 3B. The GO and KEGG analyses revealed pathways linked to these genes (Fig. 3C). GSEA identified the signaling pathways of different activated CCL11-related genes in BRCA. Results indicated significant enrichment of immune-related gene sets among CCL11-associated DEGs (Fig. 3D).

**Fig. 4:**
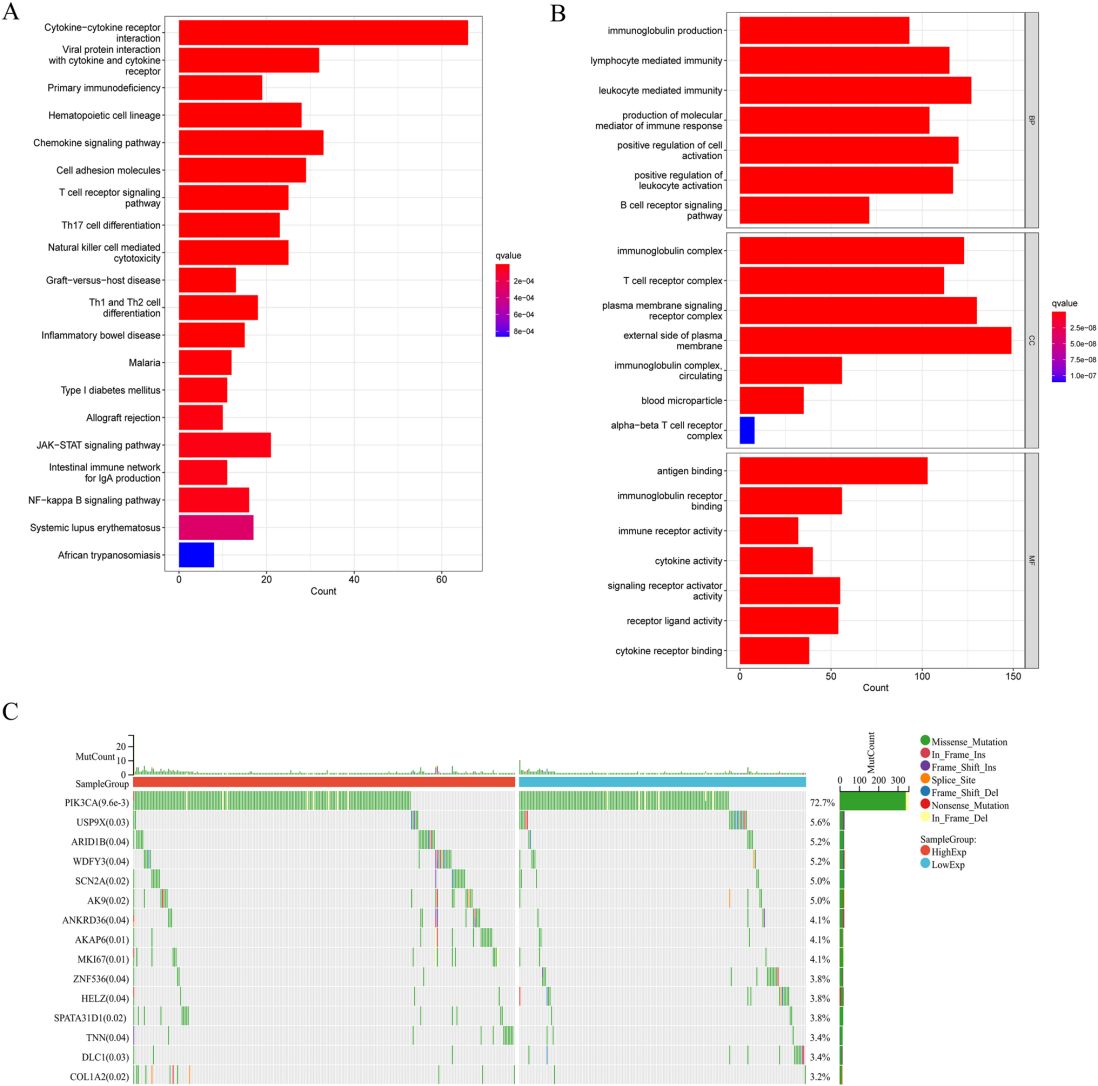
**A** GO analysis of CCL11 in breast cancer; **B** KEGG pathways of CCL11 in breast cancer; **C** The mutation profiles of genes in patients with BRCA with low and high CCL11 expressions

GO analysis revealed CCL11's involvement in the immunoglobulin production, lymphocyte mediated immunity, and T cell receptor complex (Fig. 4A). KEGG analysis associated CCL11 expression with Cytokine−cytokine receptor interaction, viral protein interaction with cytokine and cytokine receptor, and primary immunodeficiency (Fig. 4B). Patients with BRCA exhibited substantial genetic mutations between CCL11 high and low groups, including PIK3CA, USP9X, ARID1B, WDFY3, SCN2A, AK9, ANKRD36, AKAP6, MKI67, ZNF536, HELZ, SPATA31D1, TNN, DLC1, and COL1A2 (Fig. 4C).

### Analysis of co‑expression genes associated with CCL11 in BRCA

**Fig. 5:**
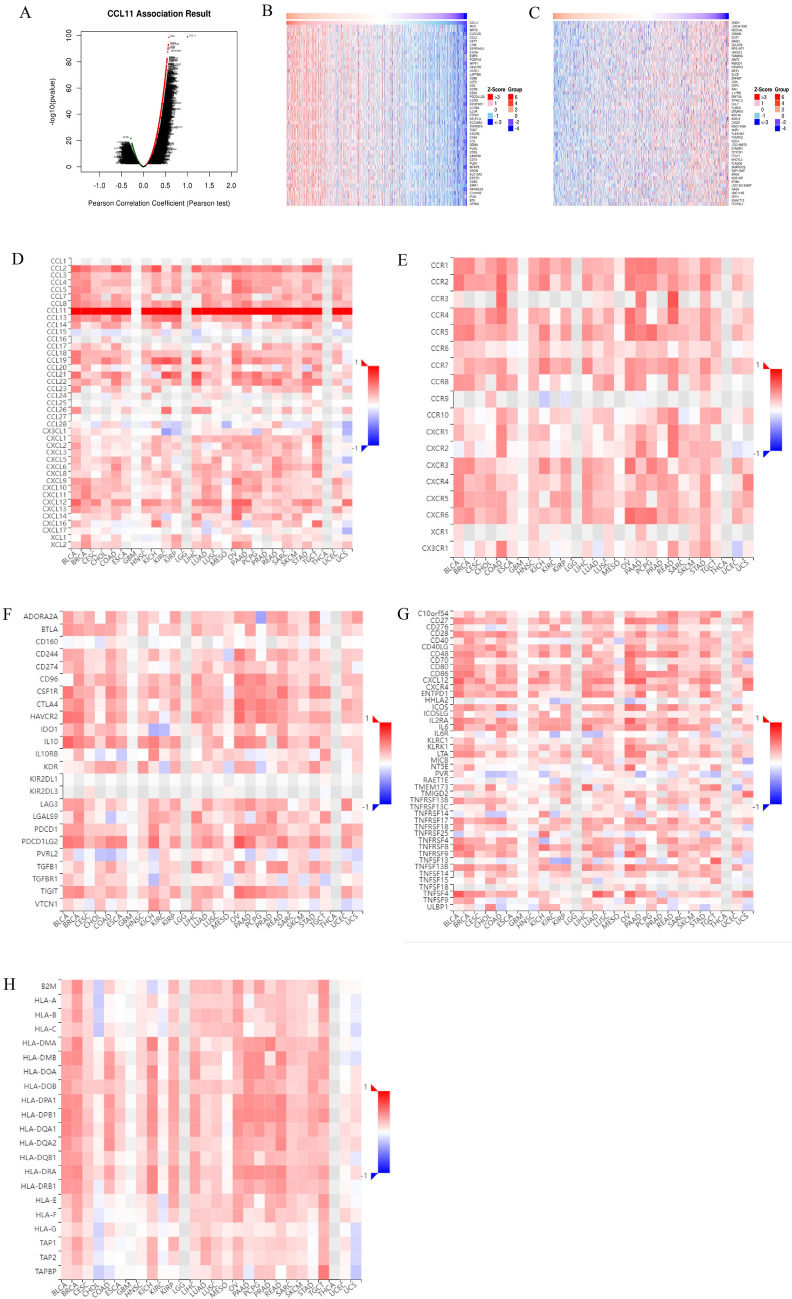
**A** The mRNA sequencing data of patients with breast cancer; **B** heat map of genes positively related to CCL11; **C** heat map of genes negatively related to CCL11; **D** association between CCL11 and immune activating genes; **E** association between CCL11 and immune inhibitive genes; **F** correlation between CCL11 and chemokines; **G** correlation between CCL11 and chemokine receptor; **H** correlation between CCL11 and MHC genes

The genes significantly related to CCL11 were shown by volcano map and thermogram through the gene co-expression module of LinkedOmics database **(**Fig. 5A–C**)**. The TISIDB database heat map showed significant positive correlations between CCL11 expression and various immune-related genes, including immune activators, suppressants, chemokines, receptors, and MHC genes in breast cancer **(**Fig. 5D–H**)**.

### Correlation between CCL11 and immunity infiltration in BRCA

**Fig. 6:**
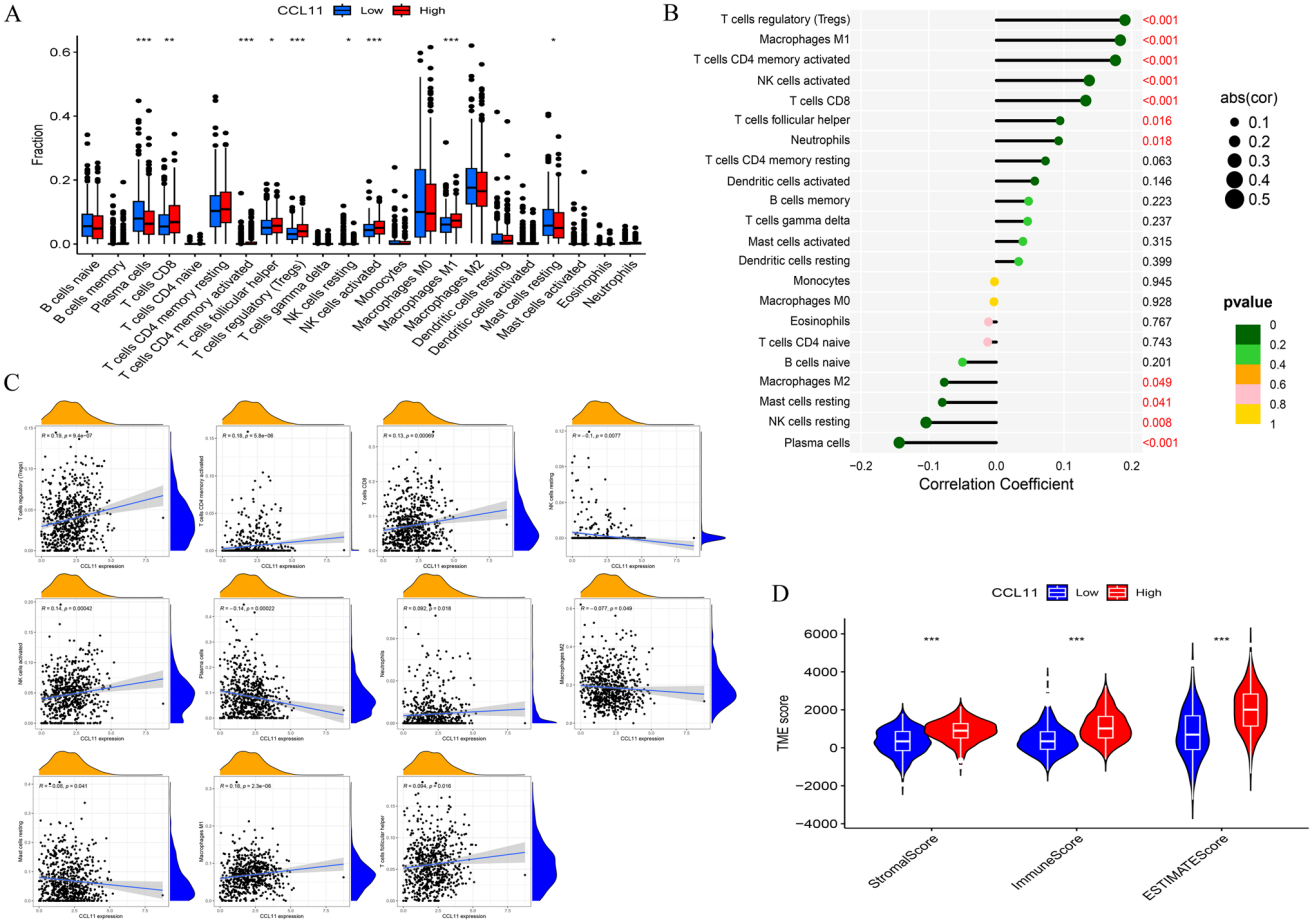
**A** The proportion of 22 immune cells infiltrating in low and high CCL11 expression groups; **B** relationships between the expression of CCL11 and 22 types of immune infiltration cells; **C**–**F** the relationships between the expression of immune infiltration cells; **G** ESTIMATE algorithm was used to investigate the correlation between the two groups in immune scores and stromal scores

CIBERSORT quantified the proportions of 22 infiltrating immune cell types in breast cancer tissues (Fig. 5A). The high CCL11 expression group showed increased levels of regulatory T cells (Tregs), memory CD4 + T cell, CD8 + T cell, Neutrophils, M1 macrophages, and follicular helper T cells (Fig. 6B). CCL11 expression was significantly negatively correlated with NK cells, plasma cells, M2 macrophages, and resting mast cells (Fig. 6C). The estimation algorithm was used to analyze the correlation between immune and stromal scores among the two groups. Higher immune and stromal scores were observed in the group with high CCL11 expression compared to the low-expression group (Fig. 6D), suggesting CCL11's influence on immune activity within the breast cancer TME.

### Correlation between CCL11 expression and drug sensitivity in BRCA

**Fig. 7:**
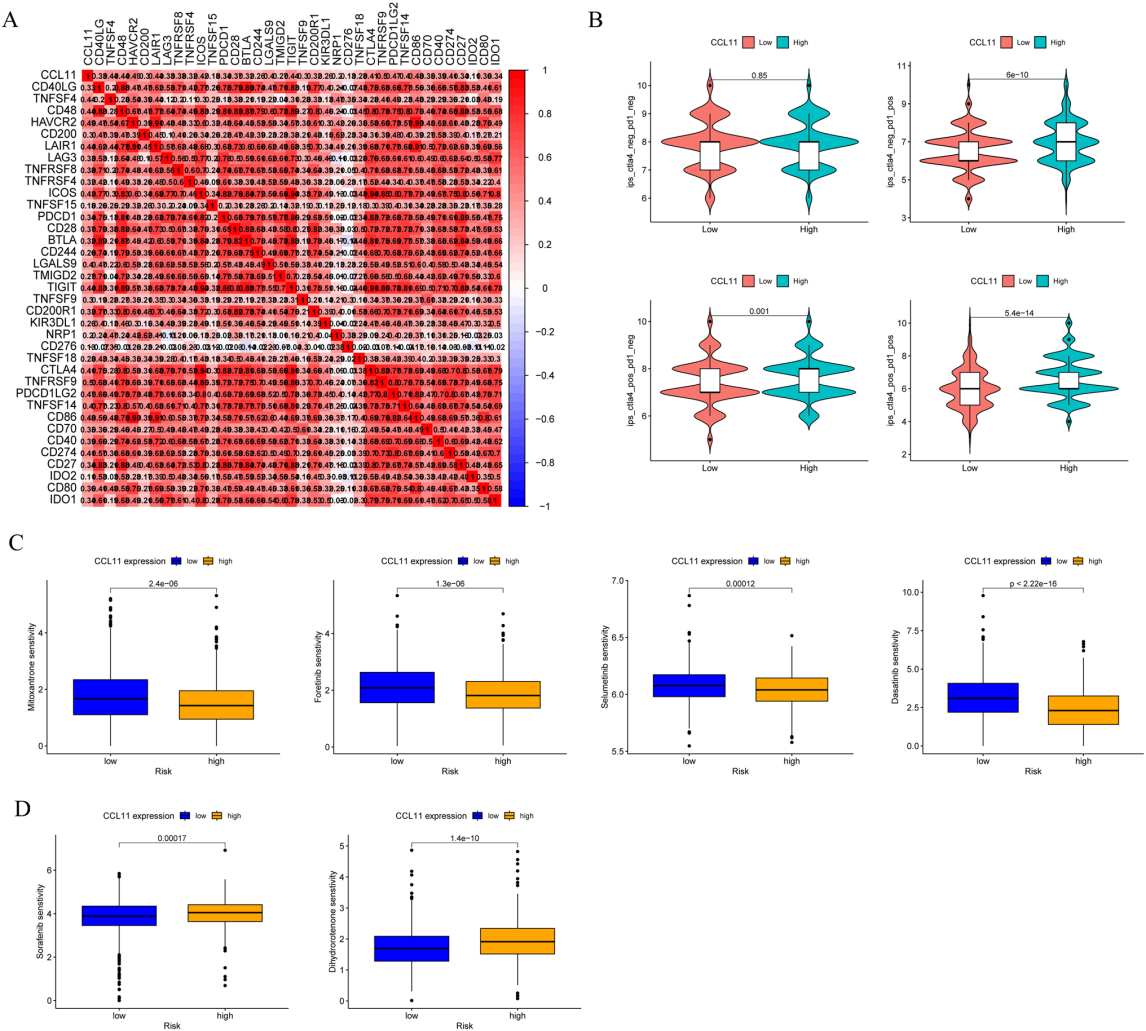
**A** The association between ICIs and CCL11; **B** the correlation between immunophenoscore and different CCL11 expression groups; **C** sensitivity analysis of mitoxantrone, foretinib, selumetinib, and dasatinib in patients with BRCA; **D** sensitivity analysis of sorafenib and dihydrorotenone in patients with BRCA

The relationship between immune checkpoint inhibitors (ICIs) and CCL11 was analyzed. We found that CCL11 was positively correlated with some ICIs, such as CD40LG, TNFSF4, and CD48 **(**Fig. 7A). Immunophenotypic analysis indicated higher IPS in the high CCL11 expression group, suggesting a more robust positive response to immunotherapy in these patients (Fig. 7B). BRCA patients with low CCL11 expression were highly sensitive to mitoxantrone, foretinib, selumetinib, and dasatinib (Fig. 7C), while those with high expression were more sensitive to sorafenib and dihydrorotenone (Fig. 7D).

### Validating of CCL11 expression in clinical samples

**Fig. 8:**
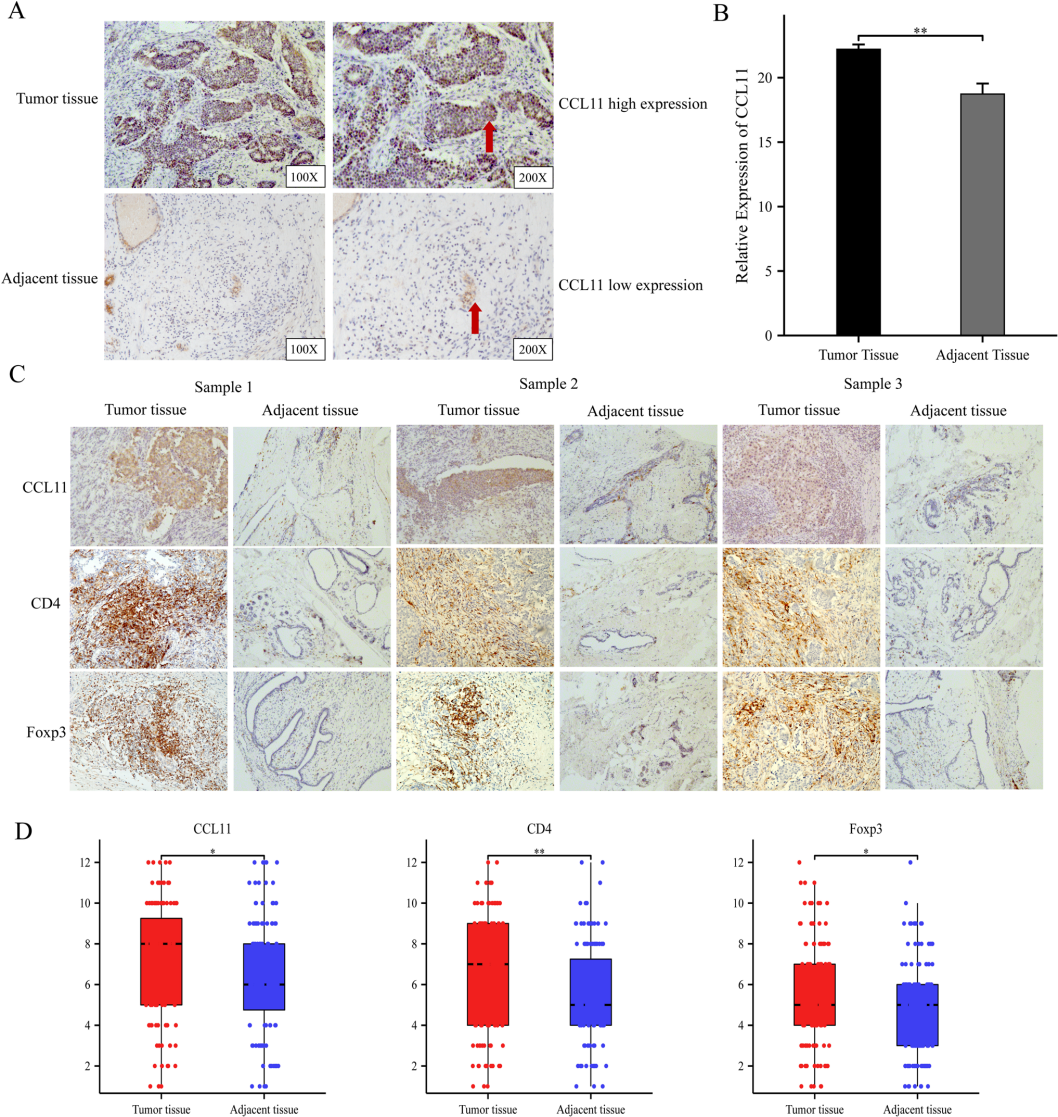
**A** CCL11 expression of breast cancer samples in our cohort through IHC; **B** CCL11 expression of breast cancer samples in our cohort through PCR; **C** Typical IHC images of CCL11, CD4, and Foxp3 proteins in the same breast cancer tissue; **D** Statistical analysis showed that CCL11, CD4, and Foxp3 in tumor tissues of the same patient were significantly higher than those in adjacent tissues

IHC staining showed higher CCL11 expression in clinical tumor samples compared to adjacent normal tissues (Fig. 8A), corroborating TCGA database findings. Using IHC images, we also assessed the association between CCL11 expression and the clinical features of BRCA patients. Statistical analysis based on tumor stage showed significant differences between CCL11 high‑ and low‑expression groups (Table 1). In addition, CCL11 was associated with molecular typing (Table 1). PCR results showed that CCL11 was significantly highly expressed in tumor tissues (Fig. 8B).

**Table 1:** Correction between CCL11 expression and clinical characteristics of patients with BRCA (*n* = 120)

Clinicopathological characteristics	Number	High Expression	Low expression	*p* value
Age (years)				0.457
≤ 50	62	30	32	
> 50	58	32	26	
Stage				0.08
Stage I	39	27	12	
Stage II	71	31	40	
Stage III	10	4	6	
T classification				0.005
T1	47	33	14	
T2	71	28	43	
T3	2	1	1	
N classification				0.855
N0	88	47	41	
N1	22	11	11	
N2	8	3	5	
N3	2	1	1	
Grade				< 0.001
1	19	17	2	
2	90	37	53	
3	11	8	3	
Subtype				0.012
Luminal A	22	14	8	
Luminal B	70	34	36	
HER-2 +	16	4	12	
Basal	12	10	2	
ER status				0.838
Positive	33	17	16	
Negative	87	43	44	
PR status				0.524
Positive	36	17	19	
Negative	84	45	39	
HER2 status				0.178
Positive	79	45	34	
Negative	41	17	24	
Ki-67 status				0.326
≤ 14	23	14	9	
> 15	97	48	49	

This study revealed a significant correlation between CCL11 expression and Treg infiltration levels. It is currently believed that CD4 + CD25 + Foxp3 + is the dominant phenotype of Treg cells. We conducted IHC staining of CCL11, CD4, and Foxp3 proteins in serial sections of the same tumor tissues from BRCA patients. Typical IHC images are presented in Fig. 8C. Expressions of CCL11, CD4, and Foxp3 were significantly increased in tumor tissues compared with adjacent tissues (Fig. 8D).

### CCL11 inhibits the proliferation, migration, and invasion of breast cancer cells, blocks the AKT pathway

**Fig. 9:**
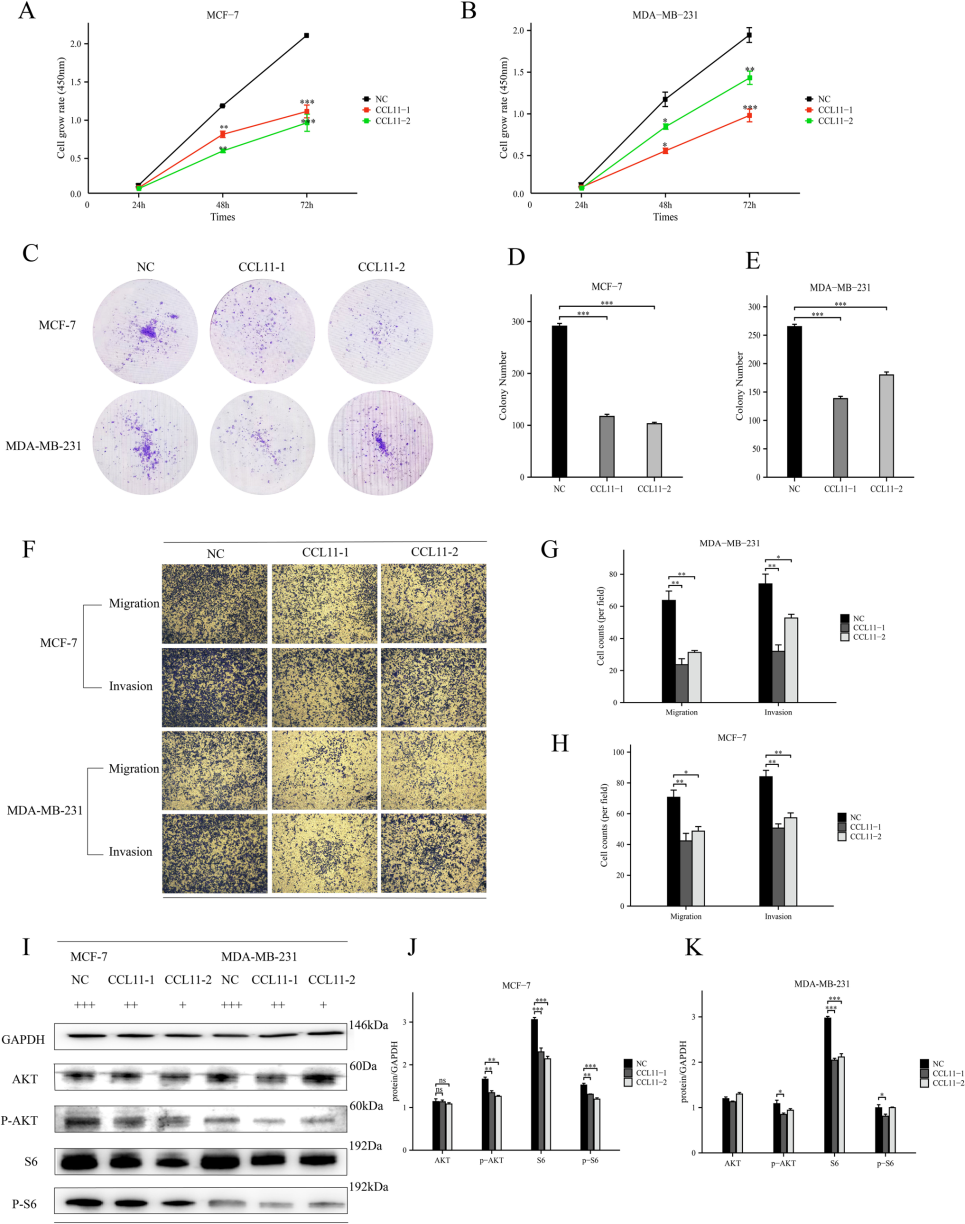
CCL11 inhibits malignant behavior of breast cancer cell lines. **A**, **B**: after adding CCL11, the proliferation capacity of breast cancer cells was measured by CCK-8; **C**–**E** after adding CCL11, the proliferation and viability of breast cancer cells were detected by plate cloning; **F**–**H** transwell assay evaluated the migration and invasion ability of breast cancer cells after adding CCL11; **I**–**K** the protein levels of AKT, p-AKT, S6, and p-S6 in breast cancer cells after addition of CCL11 were detected by Western blot

Next, we analyzed the biological function of CCL11 in breast cancer through cellular experiments. CCK-8 and colony formation experiments showed that the proliferation and colony formation of tumor cells were inhibited in vitro after the addition of CCL11 (Fig. 9A–E). We further assessed CCL11's role in breast cancer progression by examining its effects on cell migration and invasion. Transwell experiment showed that the addition of CCL11 significantly reduced the invasion and migration ability of tumor cells (Fig. 9F–H). Western blotting was conducted to analyze proteins in the PI3K-AKT pathway. Results indicated that CCL11 significantly downregulated AKT, p-AKT, S6, and p-S6 protein levels in both cell lines (Fig. 9I–K). These findings suggest a potential tumor-suppressive role for CCL11 in breast cancer.

## Discussion

An increasing body of research indicates that tumor growth is a complex process regulated by the TME (Greten and Grivennikov 2019; Laplane et al. 2019). The tumor growth process is regulated not only by its own internal signals but also by external factors which are usually active in the TME where cancer cells are located. The TME, shaped by tumor cells, can facilitate cancer development (Deepak et al. 2020). The malignant phenotype of cancer depends not only on the intrinsic activity of cancer cells but also on the components of the TME, especially the TILs (Badalamenti et al. 2019). Detailed analysis of the tumor immune microenvironment can reveal new biomarkers for prognosis and treatment prediction, significantly impacting the development of therapeutic approaches and guiding primary tumor treatment.

Studies indicate a high expression of CCL11 in breast cancer tissues, correlating with improved patient prognosis (Korbecki et al. 2020). An analysis of the TCGA database revealed significant upregulation of CCL11 in breast cancer tissues versus adjacent normal tissues. CCL11 expression is associated with BRCA's clinical features such as tumor stage, grade, and molecular subtype. PCR and IHC analysis confirmed these trends at both mRNA and protein levels. Survival analysis indicated that high CCL11 expression significantly correlates with improved survival in BRCA. Enrichment analysis demonstrated CCL11's association with multiple immune-related signaling pathways. Co-expression analysis revealed a positive correlation between CCL11 and the majority of immune-related genes. In TME of breast cancer, Tregs participate in the genesis and development of tumors by inhibiting anti-tumor immunity. Given that CD4 + CD25 + Foxp3 + is the predominant Treg phenotype (Jang et al. 2019), we utilized hospital cohort breast cancer samples for IHC testing of CCL11, CD4, and Foxp3 proteins. The results showed that the expression of CCL11 was positively correlated with the infiltration level of Tregs.

To explore the mechanism of action of CCL11 in cancers, we reviewed previous reports. CCL11 is believed to function by activating the CCR3 receptor, which enhances VEGF expression in liver cancer cells and promotes tumor angiogenesis (Park et al. 2017). Expression of CCL11 and CCR3 promotes tumor aggressiveness in head and neck cancer (Huang et al. 2022). The interaction between CCL11 and CCR3 enhances the survival of anaplastic large cell lymphoma cells via ERK1/2 activation (Miyagaki et al. 2011). Bone marrow-derived suppressor cells (MDSC), through CCL11, activate ERK/AKT signaling, inducing epithelial–mesenchymal transition and promoting lung cancer metastasis (Lin et al. 2021). A recent study discovered that CCL11 stimulation can induce eosinophil differentiation in a mouse model (Xing et al. 2016). These induced eosinophils not only damage tumor cells but also inhibit angiogenesis (Huang et al. 2022). This suggests a complex regulatory role for CCL11 within the TME of malignant tumors. Addition of CCL11 to breast cancer cells significantly inhibited proliferation, colony formation, migration, and invasion, suggesting a tumor-suppressive effect of CCL11. We also showed that CCL11 inhibits breast cancer cell carcinogenesis by modulating the AKT/S6 pathway, indicating its potential as a drug target.

Significant challenges persist in understanding breast cancer immunobiology, especially in developing reliable biomarkers and selecting immunotherapy standards. Immunotherapy, especially with immune checkpoint inhibitors, has long been known as a potentially effective treatment for breast cancer (Gaynor et al. 2022). In BRCA, studies on immune checkpoints mainly include targeting programmed cell death protein 1 (PD-1) and PD-1 ligand (PD-L1). These checkpoints block signal transduction in the negative regulatory pathway of T cell activation, thus enhancing T cell activity by reducing inhibition (Su et al. 2016). Consequently, antibodies that block immune checkpoints show considerable potential in breast cancer treatment. Nevertheless, additional research is required to support our findings, and to consider the impact of other possible factors controlling CCL11 expression. The results of survival analysis are limited by the good prognosis of breast cancer. In our cohort, there was no significant difference in OS and RFS between different CCL11 expression groups. An extended follow-up is essential to better evaluate CCL11's prognostic impact. To reinforce the transcriptomic findings, an increase in the number of clinical samples is necessary. Thus, further investigation into CCL11's role and prognostic value in the context of immunotherapy is warranted to determine its potential as a therapeutic target for breast cancer.

## Conclusions

In summary, our study demonstrated that CCL11 is a potential independent prognostic biomarker with a tumor-suppressive function in breast cancer.

## Supplementary Information

Below is the link to the electronic supplementary material.

Supplementary file1 (DOCX 15 KB)

Supplementary file2 (TIF 4366 KB)

## Data Availability

The datasets used and analyzed during the current study available from the corresponding author on reasonable request. This study has been licensed by KEGG.
