# Supplementary material for: Exploring CCL11 in breast cancer: unraveling its anticancer potential and immune modulatory effects involving the Akt-S6 signaling
Source: J Cancer Res Clin Oncol. 2024 Feb 2;150(2):69. doi: 10.1007/s00432-023-05600-6 (PMC10837270; doi:10.1007/s00432-023-05600-6)
Supplement: Supplementary file 1 — Supplementary file1 (DOCX 15 KB) [file 432_2023_5600_MOESM1_ESM.docx]

Supplementary Table 1：The primer sequences of CCL11

| **Name** | **Forward primer (5′-3′)** | **Reverse primer (5′-3′)** |
| --- | --- | --- |
| CCL11 | AGAGAGAGCGTTCCAGACAGC | AGTGCTCCACTTCCCTTCATC |
