# Supplementary figures and images for: Exploring CCL11 in breast cancer: unraveling its anticancer potential and immune modulatory effects involving the Akt-S6 signaling
Source: J Cancer Res Clin Oncol. 2024 Feb 2;150(2):69. doi: 10.1007/s00432-023-05600-6 (PMC10837270; doi:10.1007/s00432-023-05600-6)

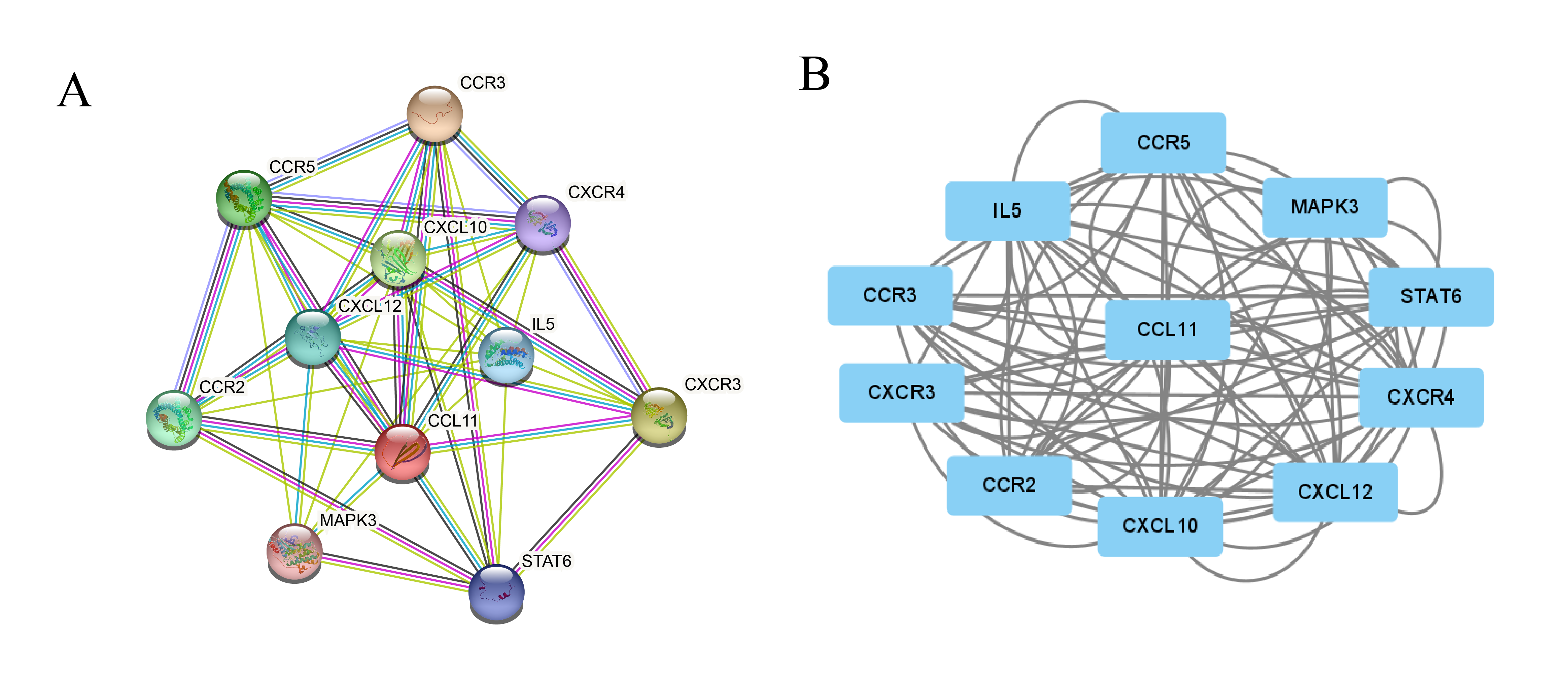

Supplement: Supplementary file 2 — Supplementary file2 (TIF 4366 KB) [file 432_2023_5600_MOESM2_ESM.tif]
